# From Traditional Medicine to Witchcraft: Why Medical Treatments Are Not Always Efficacious

**DOI:** 10.1371/journal.pone.0005192

**Published:** 2009-04-15

**Authors:** Mark M. Tanaka, Jeremy R. Kendal, Kevin N. Laland

**Affiliations:** 1 Evolution & Ecology Research Centre, School of Biotechnology & Biomolecular Sciences, University of New South Wales, Sydney, New South Wales, Australia; 2 Department of Anthropology, University of Durham, Durham, United Kingdom; 3 School of Biology, University of St Andrews, Fife, United Kingdom; Stanford University, United States of America

## Abstract

Complementary medicines, traditional remedies and home cures for medical ailments are used extensively world-wide, representing more than US$60 billion sales in the global market. With serious doubts about the efficacy and safety of many treatments, the industry remains steeped in controversy. Little is known about factors affecting the prevalence of efficacious and non-efficacious self-medicative treatments. Here we develop mathematical models which reveal that the most efficacious treatments are not necessarily those most likely to spread. Indeed, purely superstitious remedies, or even maladaptive practices, spread more readily than efficacious treatments under specified circumstances. Low-efficacy practices sometimes spread because their very ineffectiveness results in longer, more salient demonstration and a larger number of converts, which more than compensates for greater rates of abandonment. These models also illuminate a broader range of phenomena, including the spread of innovations, medical treatment of animals, foraging behaviour, and self-medication in non-human primates.

## Introduction

Traditional remedies, utilising medicinal plant and animal products, have been used as treatments for human diseases and medical conditions for millennia [1]. In recent years, 60–80% of the world's population, mainly from developing countries, depended primarily on traditional medicines, folk remedies and home cures, as well as treatment from witchdoctors and other ‘supernatural practices’, for their health-care needs [1]. In western societies, complementary and alternative medicine is garnering increasing interest and acceptance. At current growth rates, two-thirds of Americans are projected to be using alternative medicine by 2010 [2]. Asian governments are pouring billions of dollars into screening Traditional Chinese medicines in the hope that clinical trials will spawn lucrative drugs [3]. Traditional medicine has become big business.

While scientific studies have validated some traditional remedies, for instance, by confirming the biological activity of plant extracts [4], [5], the use of complementary and traditional medicines remains contentious, and doubts about the efficacy and safety of many treatments remain [1], [6], [7], [8]. Reservations over safety and efficacy underpin controversy over USA and UK universities' attempts to bring alternative medicines into medical school curricula [9]. The active ingredients used in many traditional medicines are potentially toxic, often containing dangerous elements, including heavy metals [5], [10]. Even the use of ineffective non-toxic remedies can be harmful if it delays effective treatment. For instance, fears have been expressed that, in Nigeria, witchcraft and traditional remedies of unknown efficacy are widely employed as treatments for malaria, instead of, or delaying access to, modern medicines of proven effectiveness [11]. In sub-Sarahan Africa there is a concern that the use of traditional remedies for mastitis, a condition often attributed to sorcery, may inadvertently be contributing to the spread of HIV [12].

In 2002 the WHO [1] launched a global plan to make the use of traditional medicine safer by encouraging evidence-based research on the safety, efficacy and quality of traditional practices. Accordingly, traditional medicines are currently undergoing scrutiny to evaluate their effectiveness and monitor adverse events [3], [13]. Such analyses have often failed to confirm the efficacy of traditional remedies: for instance, of nearly 25,000 applications for registration of traditional medicines received by Malaysian authorities, 37.3% were rejected, either on grounds of safety or ineffectiveness [14]. However, there is currently no compelling explanation for the prevalence of low-efficacy treatments.

Here we develop mathematical models of the spread of self-medicative treatments for medical conditions to explore the factors that lead to treatments becoming widespread, and how a treatment's efficacy affects its rate of spread. A treatment is acquired through social learning, but its spread depends on a variety of factors, including its efficacy, and the rates of conversion, death, recovery from illness and abandonment of the treatment. The approach is to derive expressions for the cultural fitness (mean number of converts to the treatment resulting directly from observation of a given demonstrator), 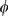, and the probability of spread of new treatments. We show that the treatments that spread are not necessarily those that are most efficacious at curing the ailment, and explain how ‘superstitious treatments’ with little efficacy and even maladaptive practices can spread under broad conditions. The models draw from two bodies of theory, cultural evolution modeling [15], [16], [17], [18] and stochastic (branching) processes [19], to develop theory applicable to investigating the spread of treatments of disease. Our analyses can also be viewed as contributing to the developing field of Darwinian medicine [20]. Although branching process models were developed to address the extinction of surnames [15], they have been more widely employed within biological evolution [21], and have yet to make further impact on the study of cultural evolution, despite extensive theory borrowing by the latter from the former [15], [16], [17], [18].

## Methods

### Basic assumptions

**Figure 1:**
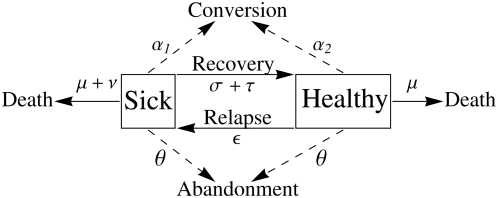
General structure of the model. This figure illustrates the processes through which demonstrators of a treatment can change health state. The parameters are defined in Methods.

The general structure of the model is illustrated in Figure 1 and the symbols used are summarised in Table 1. We assume that individuals are either in a diseased state or in a healthy state. We model the spread of a behavioural trait expressed in treatment of disease. The behavioural trait in question is any innovation, practice or treatment that could potentially affect the outcome of this disease. To model the spread of a behavioural trait, we make the following assumptions. A new behavioural trait arises in (or is invented by) an ill individual who may then *demonstrate* this practice; others who are ill may adopt the practice upon being exposed to it, and then become demonstrators themselves. In other words, demonstrators *convert* observers. There is empirical support for the assumption that self-medicative treatments spread through social learning [22]. Observers adopt the trait at a constant rate per demonstrator per unit time. This rate is 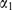 when the demonstrator is ill and 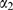 when the demonstrator is healthy. Allowing for different rates of cultural transmission from sick and well individuals is important, since treatments for many ailments, ranging from snake bites to the common cold, are primarily applied when sick, and discontinued, or practiced at a less frequent rate, when the sufferer has recovered. As our models are concerned with the initial spread of a treatment, we assume a constant supply of observers. As the dynamics of the spread of the trait are much faster than demographic changes, there are no explicit births in this model. Death, however, occurs at rate 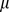 per individual per unit time; there is an additional death rate 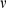 for individuals with the disease.

**Table 1:** Summary of symbols used in the model.

Symbol	Meaning
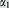	Rate of conversion of observers to the treatment practice when demonstrator is ill.
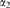	Rate of conversion of observers to the treatment practice when demonstrator is well.
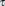	Efficacy of treatment; 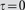 when treatment is ineffective.
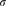	Rate of natural recovery from disease.
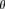	Rate of abandoning the treatment. We set 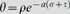 .
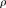	Maximum rate of treatment abandonment.
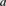	Decay in abandonment rate as efficacy increases.
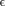	Rate of relapse to disease. We set 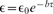 .
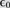	Maximum relapse rate.
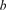	Decay in relapse rate as efficacy increases.
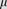	Background death rate.
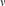	Death rate due to the disease.
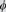	Cultural fitness of the practice (function of the parameters).
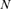	Number of observers converted by a demonstrator.
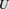	Time spent by a demonstrator being ill.
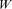	Time spent by a demonstrator being well.

Our assumption that observers adopt the trait in an unbiased fashion, and at a constant rate per demonstrator per unit time, may need further explanation. We do not assume that observers adopt self-medicative practices according to their efficacy in treating others, since we regard this to be difficult for an individual reliably to gauge. For instance, observers would be required to make a series of judgments: *Has the demonstrator the same condition as me? Is the demonstrator's judgment of its effectiveness reliable? Will the treatment work as well for me? Would the demonstrator have recovered anyway? Etc.* Rather, we leave judgments about the efficacy of treatments to self (i.e. the demonstrator), by allowing individuals to abandon the treatment, or revert to an alternative, based on their own evaluation of the treatment's effectiveness in curing themselves. Note, it does not follow from our assumption of unbiased copying that observers would be equally likely to adopt otherwise equivalent efficacious and ineffective practices, since demonstrators would be more likely to abandon the latter, as discussed below.

We are interested in both efficacious traits – those that hasten the recovery of the diseased individual – as well as those that are ineffective or even maladaptive (in that they retard or prevent recovery). The background rate of recovery is 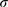 per individual per unit time, with an additional rate 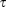 describing the efficacy of the treatment or practice (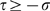). When 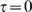 the treatment/practice is ineffective and the trait can be regarded as a superstition. When 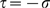 the trait is completely maladaptive because it prevents recovery, while if 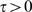 the treatment is beneficial.

**Figure 2:**
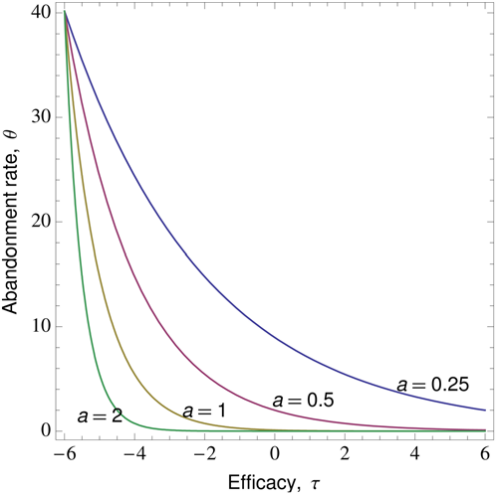
The relationship between rate of abandonment and efficacy. Here we show several curves by varying the parameter 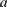 and setting 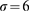 and 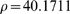 (see Methods for interpretation of parameter values).

Further assume that an individual who adopts the trait may abandon it or revert to a previous practice. The rate of abandonment is a decreasing function of the rate of recovery from the disease. This response is based on the assumption that sick individuals will become increasingly dissatisfied with their treatment as the time to recovery increases, and will abandon treatments that are perceived to be ineffective. Let this function be
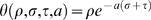
(1)where 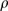 is the maximum rate of abandonment, occurring when the trait is completely maladaptive (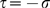), and 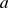 determines how strongly recovery influences abandonment (see Figure 2). While 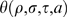 is a function of four parameters, we will write it simply as 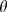 for convenience. Although we set this function to be an exponential decay, exploration of alternative forms of the relationship between abandonment and efficacy (e.g. hyperbolic function) showed they do not influence the qualitative outcomes of the analysis.

By letting recovered individuals relapse into the diseased state at rate 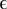 per unit time we allow for multiple episodes of illness. This dynamic is suitable for describing recurring conditions. When 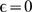 there is only a single bout of illness, a scenario we consider first in developing the models below. The rate 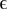 itself can be set as a function of 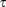, where the treatment practiced in a well state has a prophylactic effect. For example, the rate of relapse to disease might decrease exponentially at rate 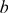 with respect to efficacy 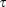. In other words, the probability that sick individuals will relapse to the diseased state decreases with increasing effectiveness of the treatment they utilise (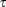).

### Constructing the model

We consider a set of special cases of the general model. In the simplest case, recovery is permanent so that there is only a single episode of illness (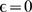), and demonstration of the treatment is restricted to the period of illness – that is, demonstration ceases upon recovery (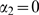). The second model generalises this situation by allowing demonstration to continue after recovery (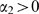), but there is still only a single episode of illness (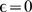). We also consider a model in which there can be multiple episodes of illness (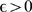) and where demonstration is restricted to sick individuals (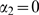). In the general case 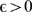 and 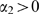.

In each model, we focus on a single demonstrator and track the total number of individuals he or she converts. This is achieved by accounting for the conversion rates 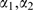 and the length of each period spent by the demonstrator being ill and well (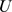 and 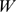 respectively). The time spent being an ill demonstrator within a given episode of illness is distributed exponentially with parameter
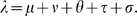
When the demonstrator is well, the time until death, abandonment or becoming ill again is distributed exponentially with parameter
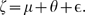
The cultural fitness of the treatment 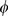 is given by the mean number of converts produced by the demonstrator. We report below the formulas for cultural fitness and provide derivations in Appendix S1. The appendix also considers the probability of spread of the treatment from the innovator, which can be derived analytically for the first model (Section A.1.1) and otherwise studied through computer simulation (Section A.2.3).

In the first model, where there is a single episode of illness and demonstration only occurs during illness, the cultural fitness is given by
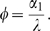
(2)


Under the second model, where there is a single episode of illness and demonstration is continued after recovery,
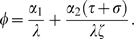
(3)


Under the most general model, where there can be multiple episodes of illness and continuous demonstration of the treatment,
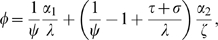
(4)where 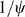 is the mean number of episodes before abandonment of treatment or death and 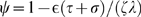. The case where there are multiple episodes but restricted demonstration is specified by setting 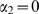 in Equation 4, giving 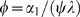.

### Parameter values

Unless otherwise specified, parameters in the numerical analysis take the following values. We assume 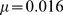 and 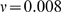 per individual per year, corresponding to a 62.5 year lifespan without disease and an illness-related death rate which is half that of natural causes. We set 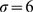, corresponding to an average episode of illness lasting 2 months. The number of converts per year per sick individual 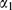 was set to 12, and that per healthy individual to 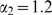. Other parameter values are: 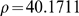, corresponding to an average time of around 0.3 months before abandonment when 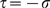 and gives a maximum cultural fitness 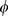 when the treatment efficacy 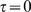; 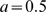, corresponding to an average time of 6 months before abandonment when 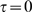 and 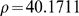; and 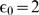, corresponding to an average time of 6 months of being healthy before relapse to disease when 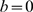.

## Results

We study our general model through subclasses, considering cases in which individuals experience either a single or multiple episodes of illness, and demonstration of the practice is either restricted to sick individuals, or continues after recovery.

**Figure 3:**
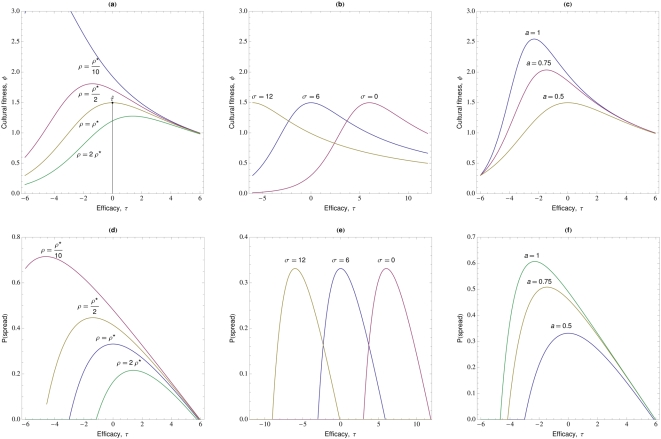
A single episode of illness and demonstration only during illness. The cultural fitness (a–c) and probability of spread (d–f) of self-medicative treatments, plotted as a function of treatment efficacy, 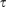, when there is a single episode of illness and demonstration occurs only during illness. Left (a and d), effect of varying maximum rate of abandonment, 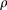. Middle (b and e), effect of varying rate of recovery, 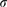. Right (c and f), effect of varying rate of decay in treatment abandonment. Unless otherwise stated 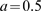, 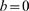, 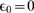, 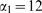, 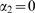, 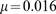, 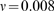, 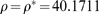 and 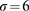 (see also Methods and Table 1).

First consider cases with a single bout of illness and treatment demonstration restricted to sick individuals (Equation (2), Figure 3). Across a broad range of conditions, the most efficacious treatments are not necessarily those most likely to spread, and superstitious treatments with no efficacy (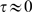), or even maladaptive practices (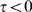), frequently have the highest cultural fitness (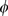). Superstitious treatments and maladaptive practices can spread because their very ineffectiveness results in sick individuals demonstrating the practice for longer than efficacious treatments, leading to more salient demonstration and more converts. This outcome occurs in spite of the fact that we assume that the less effective the treatment, the more likely a sick individual will abandon it, resulting in n-shaped functions for cultural fitness (Figure 3a–c) and probability of spread of the treatment (Figure 3d–f) The observed relationships represent a trade-off between duration of illness which is associated with demonstration of the treatment on one hand and retention of the treatment due to its efficacy on the other hand. That is, persistent illness leads to prolongued demonstration of the practice, yet an increased rate of abandonment of an ineffective treatment. In contrast, increased retention of an effective treatment is also associated with reduced demonstration of the practice.

The quality of treatments that successfully spread depends critically on the rates of recovery from illness and abandonment of the treatment, with high-recovery/low-abandonment favouring superstitious/maladaptive treatments, and low-recovery/high-abandonment favouring efficacious treatments (Figure 3). From an evolutionary perspective, suppose the treatment or practice evolves through competition between alternative forms, each with its specific efficacy. Assuming treatments of higher cultural fitness always displace those of lower cultural fitness, the evolutionarily stable strategy in this system may well be a maladaptive treatment that hinders recovery. This scenario occurs when the abandonment parameter 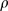 is sufficiently low relative to the recovery rate 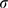. Intuitively, this case describes a situation where individuals are very persistent in using a treatment, resulting in the spread of poor practices in the long term. Factors that precipitate low abandonment, such as social norms favouring traditional remedies, or treatments that are costly to learn, potentially facilitate the spread of superstitions/maladaptive traits, particularly in chronic cases. This analysis can explain the ineffectiveness of many prominent complementary and traditional medicines.

**Figure 4:**
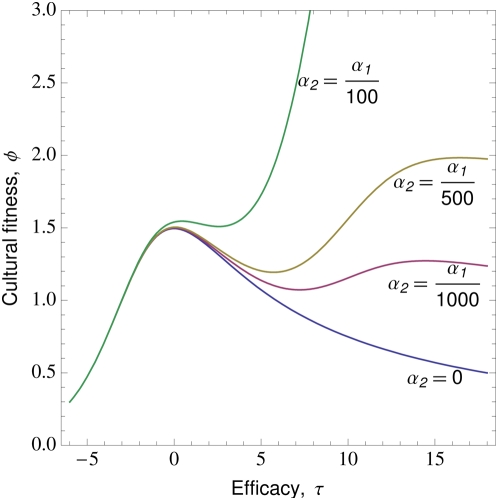
A single episode of illness and continued demonstration. The cultural fitness of self-medicative treatments (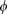) plotted as a function of treatment efficacy, 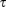, when there is a single episode of illness (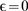) and demonstration continues after recovery (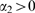). Parameter values are 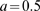, 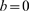, 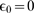, 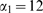, 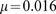, 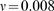, 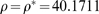 and 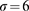 (see Methods for interpretation of parameter values).

Continued demonstration after recovery (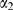 positive) typically increases the probability that efficacious treatments will spread, by weakening the aforementioned trade-off between retention of treatment and duration of illness, because a fast recovery does not prevent subsequent recruitment of others to the practice (Figure 4). If the conversion rate after recovery is sufficiently high relative to that in sickness, efficacious treatments are more likely to spread than maladaptive/superstitious treatments. Numerical analysis leads to the general prediction that, other factors being equal, treatments solely demonstrated in sickness are typically less effective than treatments also demonstrated in wellness.

**Figure 5:**
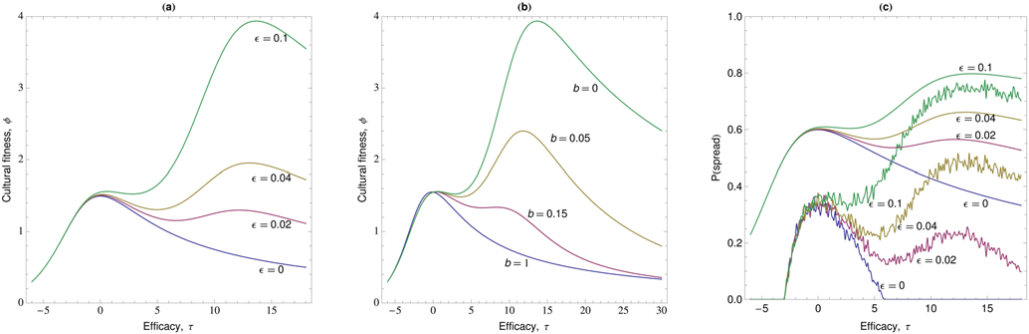
Multiple episodes of illness and demonstration restricted to sick individuals. The cultural fitness of self-medicative treatments 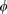, (left and middle), and probability of spread of treatments (right) plotted as a function of treatment efficacy, 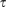. Left (a): cultural fitness 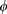; we set 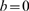 so that 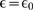. Middle (b): cultural fitness 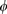; when treatment is prophylactic (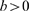). Right (c): The ultimate probability of spread (rugged lines) and the probability of spread from an innovator (smooth lines) for various rates of relapse 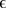 (indicated by colour). Unless stated otherwise, parameter values are 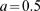, 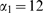, 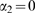, 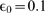, 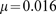, 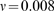, 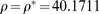 and 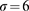 (see Methods for interpretation of parameter values).

Multiple episodes of sickness typically favour efficacious treatments, and make it more likely in general that treatments will spread compared to single episodes (Equation (4), Figure 5a). Multiple episodes allow demonstrators of efficacious treatments repeated opportunities to convert others to the practice. High efficacy, by enhancing recovery, increases the number of cycles of demonstration, weakening the trade-off between retention of treatment and duration of illness. Even with demonstration restricted to sick individuals, the efficacy of the treatment with the highest cultural fitness (or probability of spread) is typically high in cases where there is a high rate of relapse into sickness (i.e. large 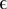).

Prophylactic treatments (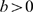) disproportionately reduce the relapse rate of efficacious traits over maladaptive treatments, thereby decreasing their opportunity to acquire new converts (for 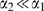). It follows that prophylactic self-medicative treatments should spread less readily than non-prophylactic treatments (Figure 5b). Figure 5c shows that the ultimate probability of spread across 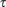 exhibits a similar pattern to that of the cultural fitness (Figure 5a,b) and that naturally, the probability of initially spreading from the inventor is always higher than the ultimate probability of spread.

**Figure 6:**
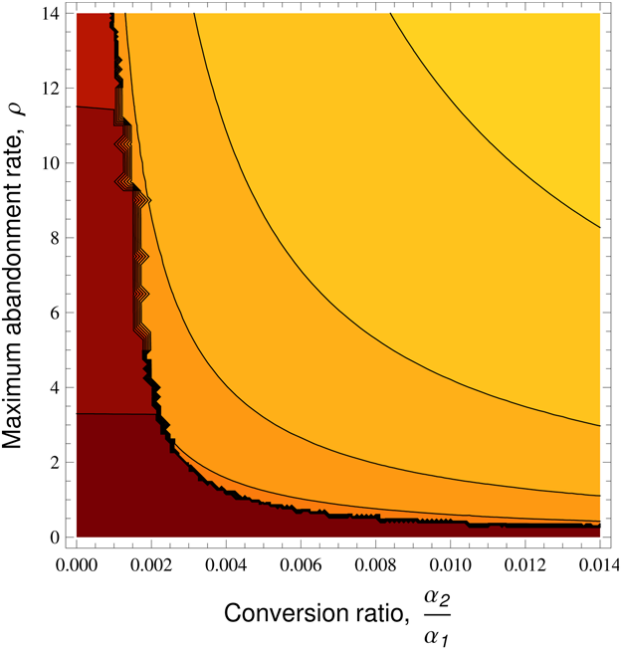
The effect of abandonment and conversion rates on the probability of spread. A density plot showing the treatment with the highest probability of spreading 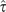 as a function of 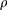 and the relative rate of conversion during healthy and sick periods (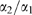), colour boundary range {−5,17.5;±2.5} (low values, dark). Unless otherwise stated 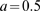, 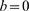, 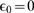, 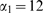, 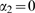, 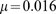, 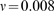, 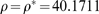 and 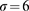 (see Methods for interpretation of parameter values).

Generally, highly efficacious treatments have higher cultural fitness than superstitious/maladaptive traits in multiple-episode cases, but nonetheless superstitious treatments (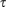 close to 0) can spread. Superstitious and maladaptive practices are most likely to spread where treatments are primarily demonstrated in sickness (i.e. a low ratio of 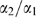) and low abandonment (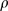), particularly where relapse is unlikely (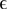 small). Figure 6 illustrates this principle through a density plot of the 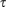 with highest probability of spread as a function of 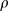 and the relative rate of conversion during healthy and sick periods (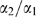).

## Discussion

This study offers a simple, novel and counter-intuitive hypothesis for the prevalence of ineffective medical treatments: unbiased copying of new treatments can frequently lead to the prevalence of ineffective practices because such treatments are demonstrated more persistently than efficacious alternatives, even when there is enhanced abandonment of ineffective cures. By unbiased copying, we mean copying in direct proportion to the rate at which the alternative variants are demonstrated. Here, in simple terms, treatment frequency dynamics are typically dominated by two processes, representing the rates of acquisition and loss of remedies. Maladaptive and superstitious treatments can become prevalent because their ineffectiveness prolongs illness, enhancing their rate of demonstration relative to efficacious cures, and leading to elevated rates of acquisition that may compensate for greater loss.

Our finding that superstitious treatments can easily spread is supported by reports of extraordinary treatments for conditions such as leprosy (treated with a drink made of rotting snakes) and syphilis (treated by eating a vulture), and by similar myths for poisonous snake bites (apply ‘guaco’ leaves, poisonous lizard skin or snake's bile), dog bites (drink tea made from the dog's tail) and scorpion stings (tie a scorpion against the stung finger) [23]. The analysis also helps explain the persistence of medical treatments of animals, such as ‘firing’ (cautery) of working horses, employed for millennia as treatment for lameness, where recovery is rare, and still widely practiced in many countries in spite of trials establishing its ineffectiveness [24]. In such cases, of course, the treatment belief is acquired by the owner, rather than by the diseased individual.

Even when highly effective treatments have higher cultural fitness values than ineffective treatments, our analysis shows that such highly functional innovations can easily be lost due to stochasticity. This has not been apparent to researchers studying the diffusion of foraging innovations in animals, for whom the failure of most innovations to spread, particularly those beneficial to the inventor, has been regarded as a mystery [25]. In fact, the observation that the majority of beneficial innovations are frequently lost is exactly what our model predicts.

The analyses presented here could usefully be extended to model disease frequencies explicitly, and to incorporate the costs of treatment. It is well-established that human life-history decision making is affected by costs [26], and this is also likely to be true of medical-treatment decisions. Nonetheless, our models make sense of a surprisingly broad set of phenomena.

### Applications of the model

The primary application of our models is to the spread of self-medicative treatments in humans. The models are potentially relevant to any socially learned practice that is thought by the user to affect (that is, treat) their medical condition, through aiding recovery, reducing suffering, or reducing the probability of relapse, *irrespective of whether or not the treatment actually does bring about the improvements in condition assumed by the user*. The treatments include modern/established medical practices, complementary medicines, traditional medicines and alternative medicines. The models also potentially apply to instances of witchcraft, shamanism and magic in which the ‘treatment’ is believed to combat perceived underlying ‘supernatural’ causes of disease (e.g. the curse of a jealous neighbour, or a haunting by the ghost of an ancestor), so long as there exists a physical ailment in the user, and the treatment propagates through cultural transmission. While the use of modern medicines and well-documented and established complementary treatments is ontologically distinct from witchcraft and shamanism (they are not substitutes for each other, and their effectiveness is likely to be gauged by different criteria), nonetheless, all of these treatments share the fact that their use spreads through social learning and transmission, and uptake therefore is potentially a function of the rate of practice demonstration. Relevant medical conditions include physical and psychological disease, injuries and accidents. While the model can be applied to treatment of some infectious disease, we note that a satisfactory analysis of medical conditions that propagate rapidly relative to the rate of spread of treatments would require an extension of these models to track disease spread explicitly.

While the models assume treatments spread through social learning, the precise nature of the psychological mechanism is unspecified, and any of a range of established processes could be operating [27], [28]. Nor do the models require the *conscious* imitation or observational learning of a practice. Accordingly, while the models are developed with humans in mind, they potentially are relevant to the spread of self-medicative treatments in other animals, particularly nonhuman primates. There is now good evidence for self-medicating behaviour in nonhuman primates, particularly African apes [29], [30]. Chimpanzees, bonobos and gorillas are known to swallow whole and defecate intact leaves, traditional behaviour thought to be a means of purging intestinal parasites [29], [30]. Experimental evidence reveals that this can spread through social transmission, leading to the suggestion that self-medicative practices in apes are maintained as behavioural traditions [29], [30], [31]. As intestinal parasites are likely to inflict multiple episodes (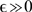), circumstances that should favour the spread of efficacious treatments, our analysis supports claims of effective self-medication in apes.

The models are also potentially applicable to veterinary practices, although here the sick/treated animal is a different individual to the individual practicing the treatment (its owner). The models are not applicable to the activities of a veterinarian, but rather to animal owners who apply socially transmitted knowledge to treat their animal's condition.

The models apply broadly to any case where there are two states associated with higher and lower mortality, with the behavioural practice affecting the transition from the former to the latter. For instance, the model could be used to investigate the diffusion of foraging innovations, where hunger equates to sickness and satiation to wellness. With ‘multiple episodes’ of hunger (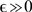), a high rate of abandonment of poor foraging techniques or low profitability foods (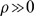), and no ‘recovery’ without feeding (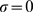), our model predicts that efficacious traits are most likely to spread, and that the frequency of maladaptive or superstitious foraging innovations should be low. This conclusion holds even if alternative foraging strategies were available (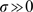), as would be the case where animals can feed without requiring social information. This may help to explain why there is not the same controversy over the spread of alternative foods and food-processing techniques as there is for treatments of disease: the latter are significantly more likely to be ineffective.

### Remarks on unbiased copying

Our choice to set copying to be unbiased is a simple and parsimonious assumption. We also believe it is close to reality. Indeed, in recent years, considerable evidence has accumulated for such unbiased copying in the transmission of a broad range of cultural traits, from pottery designs, to baby names, to the popularity of dog breeds [32], [33], [34], but our analysis extends these findings to cultural traits that potentially affect Darwinian fitness. While individuals may seek to acquire effective remedies, they typically fail to do so in practice. In many circumstances, making judgments about the effectiveness of treatments deployed by others is challenging. For most ailments and practices, the decision to adopt a treatment is based on weak circumstantial evidence, cultural preconceptions and perceived efficacy, which may not reflect actual efficacy. Cultural mileux that frame natural phenomena in terms of supernatural causes would further weaken the connection between efficacy and the rate of adoption. Moreover, it is likely that people frequently recover irrespective of treatment (i.e. 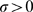), are poor at making judgments about what led to recovery, and different people offer conflicting advice. Our model therefore makes the simple assumption that sick people are willing to try new remedies – through unbiased copying – and drop them if they do not appear to work, and this suffices to explain superstitious and maladaptive treatments. We note that if copying were strongly biased so that individuals adopt effective treatments preferentially over ineffective ones, both acquisition and loss processes would favour effective remedies, leading to the spread of only efficacious treatments. At best, such a scenario could account for the presence of adaptive remedies, and could not by itself explain the existence of maladaptive or superstitious treatments. Yet, as described above, there is strong evidence that ineffective treatments are commonplace [1], [6], [7], [8], [11], [12], [13], [14].

Unbiased copying should be distinguished from another notion – that sick people, in desperation, are willing to try any available treatment. Such a practice would not bring about unbiased copying (acquisition in proportion to observed frequency), but rather frequency independent copying, since all treatments would be equally likely to be adopted, irrespective of their frequency or efficacy. With no acquisition bias, treatment frequency dynamics would be dominated by the loss-process, which favours effective cures due to the abandonment of ineffective treatments. While the ‘desperate flailing’ process would preserve variation in treatments at low level, it could not explain how maladaptive or superstitious treatments could reach high frequencies. Moreover, this hypothesis runs counter to the strong empirical evidence that social learning increases with the frequency of demonstration [35], [36], [37], [38], [39]

Conceivably, in humans, the trade-off between trait efficacy and probability of spread predicted by our models will sometimes be negated through language. Individuals can simply sample others' evaluations, for instance, through conversation. However there is theoretical and empirical support for the hypothesis that individuals preferentially evaluate appropriate behaviour based on direct cues (e.g. self-evaluation), rather than observed behavioural decisions of others (e.g. evaluating others' treatments) [40], [41], [42]. Here, the contrast between technological/foraging innovations and medical treatments may be instructive. For technological/foraging innovations, the productivity of the innovation when employed by others is likely, in many situations, to be relatively straightforward to gauge, since observers can directly see the returns (higher yield, a better product etc) and make reliable judgments. This will mitigate against the spread of arbitrary of maladaptive practices, since observers would no longer be copying at random, but according to the efficacy of the trait. Individuals could equally inform others of effective self-medicative treatments, but with lower reliability, since the aforementioned factors (the difficulties of determining similarity of condition, demonstrator reliability, equivalence of treatment and the fact that individuals may have recovered independent of treatment), together with cultural norms about how medical conditions should be treated (e.g. the local convention in sub-Saharan Africa is to treat mastitis with witchcraft [12]) and placebo effects that accelerate recovery even when biologically inactive treatments have been adopted, render impartial evaluation of efficacy difficult.

### Alternative hypotheses

While several established cultural evolution models explain the persistence of maladaptive traits, none are credible alternative explanations for the existence of ineffective or maladaptive self-medicative treatments on the scale observed. Selfish cultural variants, or memes [43], can lead to maladaptive traits spreading if the rate of imitation exceeds that of competing adaptive variants and overwhelms opposing selection [15], [16]. To explain maladaptive treatments, however, this hypothesis would require people to prefer treatments that do not work over treatments that do, which is implausible. Conformist biases are known sometimes to lead to maladaptive outcomes, where environmental change renders a once adaptive solution no longer adaptive, or if conformity favours group-beneficial traits [16], [38]. Yet for many complementary medicines (e.g. the ‘healing’ power of crystals) there is no evidence that these treatments ever worked, nor any suggestion that they are group beneficial. Sexual selection, operating at genetic, cultural, or gene-cultural levels, is also known to be capable of propagating maladaptive variants [44], [16], [45] but people typically do not adopt medical treatments to render themselves attractive to the opposite sex. Prestige biases [16], [46] are more credible, particularly in small scale pre-industrial societies, but in modern western societies where there is considerable prestige associated with doctors and the medical establishment, these institutions have typically lobbied against the use of complementary medicines and traditional treatments. These treatments appear to have spread in spite of a counteracting prestige bias, rather than because of one. In contrast to the above, the unbiased copying explanation that we favour is both simple and plausible.

## Supporting Information

Appendix S1 (0.10 MB PDF)
