# Supplementary material for: From Traditional Medicine to Witchcraft: Why Medical Treatments Are Not Always Efficacious
Source: PLoS One. 2009 Apr 15;4(4):e5192. doi: 10.1371/journal.pone.0005192 (PMC2664922; doi:10.1371/journal.pone.0005192)
Supplement: Appendix S1 — (0.10 MB PDF) [file pone.0005192.s001.pdf]

Supporting Information S1
Appendix to
From traditional medicine to witchcraft: why medical treatments are not always efficacious

Mark M. Tanaka
Evolution & Ecology Research Centre and
School of Biotechnology & Biomolecular Sciences,
University of New South Wales, Sydney NSW 2052, Australia

Jeremy R. Kendal
Department of Anthropology, University of Durham,
Durham DH1 3HN, United Kingdom

Kevin N. Laland
School of Biology, University of St Andrews,
Fife KY16 9TS, United Kingdom

A Details of the model

This appendix section describes the mathematical details of the stochastic model of the spread of culturally transmitted traits. In particular, we derive Equations (2)–(3) in Methods.

A.1 Single episode of illness

We begin with the special cases of the model in which there is only a single episode of illness. This situation is specified by setting $\epsilon = 0$.

A.1.1 Disease-restricted demonstration

We first consider a model in which demonstrators stop their treatment or practice when they recover, and consequently observers are not converted by recovered individuals. This case is specified by letting $\alpha_2 = 0$.

If each observer has the same probability of being converted, the number of conversions is binomially distributed. If the number of available observers is not too

small, this distribution is well approximated by a Poisson. Thus, given U the time spent being ill, N is distributed as a Poisson with parameter $\alpha_1 U$. As described in Methods, U in turn is distributed exponentially with parameter $\lambda = \mu+\nu+\theta+\tau+\sigma$. Because N is a Poisson-exponential mixture, it has a geometric distribution with parameter

$$\frac{\lambda}{\lambda+\alpha_1} = \frac{\mu+\nu+\theta+\tau+\sigma}{\alpha_1+\mu+\nu+\theta+\tau+\sigma}$$

(see for example reference [1]). This can be seen through the following, letting $f(u)$ represent the probability density function of the exponential distribution:

$$\begin{aligned} P(N=n) &= \int_0^\infty P(N=n|U=u)f(u)du \\ &= \int_0^\infty \frac{e^{-\alpha_1 u}(\alpha_1 u)^n}{n!}\lambda e^{-\lambda u}du \\ &= \frac{\lambda}{\alpha_1+\lambda}\left(\frac{\alpha_1}{\alpha_1+\lambda}\right)^n \qquad \text{[integrate by parts } n \text{ times]} \end{aligned}$$

where $n = 0, 1, 2, ...$

Cultural fitness of the trait The cultural fitness of the trait in this model is the mean number of converts per demonstrator. This is analogous to the concept of the basic reproductive value in epidemic theory and absolute fitness in evolutionary theory. The mean of the geometric distribution above is

$$\phi = E(N) = \frac{\alpha_1}{\alpha_1+\lambda}\bigg/\frac{\lambda}{\alpha_1+\lambda} = \frac{\alpha_1}{\mu+\nu+\theta+\tau+\sigma}. \tag{1}$$

Probability of spread of new trait The probability of the practice being spread to at least one individual after its invention is

$$1 - P(N=0) = P(N \geq 1) = \frac{\alpha_1}{\alpha_1+\lambda}.$$

The probability of spread through the whole population, that is, fixation, is one minus the probability of (eventual) extinction. To derive the ultimate extinction probability, we use a result of linear birth-death processes [2]. Here, the linear stochastic birth rate is α_1 which is the rate of converting observers to the treatment, and the death rate is λ. The extinction probability [2, p147] is then

$$\frac{\lambda}{\alpha_1}$$

if $\lambda < \alpha_1$ and 1 if $\lambda \geq \alpha_1$. The probability of spread of the trait is therefore

$$P(\text{spread}) = \max\left(0, \frac{\alpha_1-\lambda}{\alpha_1}\right). \tag{2}$$

This result can be alternatively derived by considering the process as a discrete time branching process. In this case, the extinction probability is the smallest solution of x in

$$x = \sum_{n=0}^{\infty} x^n P(N=n),$$

giving

$$x = (1-p)/(1-xp)$$

where $p = \frac{\alpha_1}{\alpha_1+\lambda}$ and therefore

$$px^2 - x + (1-p) = 0$$

and

$$P(\text{extinction}) = \min(1, (1-p)/p) = \min(1, \lambda/(\alpha_1)).$$

The probability of spread of the trait is then as given in Equation (2). The reason a discrete formulation is possible here is that the probabilities of events do not change over time (for each individual), and thus to compute the extinction probability it does not matter whether or not the "generations" are synchronised.

Note, in this case, the relationship between the cultural fitness of the trait and the probability of its spread: if the cultural fitness is ϕ, the probability of spread is $1 - 1/\phi$ for $\phi > 1$ and 0 otherwise.

Optimal efficacy of trait Does a treatment with the highest efficacy τ have the highest cultural fitness? In other words, what efficacy maximises the cultural fitness of the behavioural trait? Under this model, the probability of spread (as well as cultural fitness) is an "n-shaped" curve. Thus, intermediate efficacy is favoured. The reason is that on one hand if the treatment efficacy is too low, the practice is abandoned because recovery is too slow, and on the other hand if it is too high, recovery is fast and opportunities for demonstrating the practice to others are reduced. Inspecting Equation (1), this qualitative result clearly holds as long as the abandonment rate increases with decreasing recovery rate. Figure 3 shows this relationship for alternative values of σ, ρ and a.

The optimal efficacy for this model occurs at

$$\hat{\tau} = \frac{\ln(a\rho)}{a} - \sigma.$$

This optimal efficacy is positive when

$$\rho > \frac{e^{a\sigma}}{a}.$$

In other words, if $\rho < e^{a\sigma}/a$ the treatment efficacy with the highest cultural fitness is negative — that is, the optimal treatment is maladaptive.

A.1.2 Continued demonstration

Here we consider a second model which generalises the above model in that demonstration can occur after an individual has recovered. Again, recovery is permanent with $\epsilon = 0$. Note that the probability that the demonstrator recovers rather than dying or abandoning the practice is given by

$$\frac{\tau + \sigma}{\lambda}.$$

Again, N is the number of observers converted by the demonstrator and U is the time spent by the demonstrator being sick until recovery, death or abandonment.

Let W be the time between recovery and death or abandonment if the demonstrator recovered. We assume U and W are independent.

Because the sum of two independent Poisson variables is also Poisson, the conditional distribution of N given the periods U and W is

$$N|U, W \sim \text{Poisson}(\alpha_1 U + \alpha_2 W)$$

where

$$U \sim \text{Exp}(\lambda),$$
$$W = \begin{cases} 0 & \text{with probability } 1 - (\tau + \sigma)/\lambda \;\; \text{(death or abandonment)} \\ W_E & \text{with probability } (\tau + \sigma)/\lambda \;\; \text{(recovery)} \end{cases}$$

and

$$W_E \sim \text{Exp}(\zeta)$$

where, in this case, $\zeta = \mu + \theta$.

Cultural Fitness of the trait Again, the expectation of the convert distribution gives the cultural fitness ϕ of the trait. Noting that $E(W) = E(W_E)(\tau+\sigma)/\lambda$, we write

$$\begin{aligned} \phi &= E(N) = E(E(N|U, W)) = \alpha_1 E(U) + \alpha_2 E(W) \\ &= \frac{\alpha_1}{\lambda} + \frac{\alpha_2(\tau + \sigma)}{\lambda\zeta}, \end{aligned} \tag{3}$$

or written out in full,

$$\begin{aligned} \phi =& \frac{\alpha_1}{\mu + \nu + \rho e^{-a(\sigma+\tau)} + \tau + \sigma} + \\ & \frac{(\tau + \sigma)\alpha_2}{(\mu + \rho e^{-a(\sigma+\tau)})(\mu + \nu + \rho e^{-a(\sigma+\tau)} + \tau + \sigma)}. \end{aligned}$$

Note that the cultural fitness of the trait is the sum of two components corresponding to the periods of being ill and being healthy.

A.2 Multiple episodes of illness

In this section we turn to the most general model in which an individual can become ill more than once, and demonstration continues after recovery. Consider the distribution of the number of episodes of illness. Let ψ be the probability that the process ends through death or abandonment in a given episode or in the period of recovery immediately following the episode. This probability is given by

$$\psi = \left(1 - \frac{\tau + \sigma}{\lambda}\right) + \frac{\tau + \sigma}{\lambda}\left(1 - \frac{\epsilon}{\zeta}\right) = 1 - \frac{\epsilon(\tau + \sigma)}{\zeta\lambda}.$$

Let H be the total number of episodes of illness experienced by an individual. This random number is distributed geometrically; that is, $P(H = i) = (1 - \psi)^{i-1}\psi$ for $i = 1, 2, 3, \ldots$.

In this model, U is the total time spent by a demonstrator being ill, which is the sum of periods of time of each episode of illness. Assuming these periods are independent and identically distributed as exponential with parameter λ, the sum, conditional on H, has a gamma distributed

$$U|H \sim \text{Gamma}(H, 1/\lambda),$$

with

$$H \sim \text{Geometric}(\psi).$$

Similarly, the total time spent being a healthy demonstrator (after the first episode of illness) is

$$W|H = \begin{cases} W_{H-1} & \text{with probability } 1 - (\tau + \sigma)/\lambda \\ W_H & \text{with probability } (\tau + \sigma)/\lambda \end{cases}$$

where $W_H \sim \text{Gamma}(H, 1/\zeta)$ and $W_{H-1} \sim \text{Gamma}(H-1, 1/\zeta)$. The two cases above correspond to death or abandonment occuring during the last phase of illness (W_{H-1}) and during the last phase of healthiness (W_H). Finally, as before,

$$N|U, W \sim \text{Poisson}(\alpha_1 U + \alpha_2 W).$$

The unconditional distribution of N therefore has the probability mass function

$$P(N = n) = \sum_{h=1}^{\infty} Ge(h; \psi) \Bigg[(1 - q) \int_0^{\infty} \int_0^{\infty} Ga(u; h, \lambda) Ga(w; h-1, \zeta) Po(n; \alpha_1 u + \alpha_2 w) du\, dw + q \int_0^{\infty} \int_0^{\infty} Ga(u; h, \lambda) Ga(w; h, \zeta) Po(n; \alpha_1 u + \alpha_2 w) du\, dw \Bigg] \quad (4)$$

where

$$q = \frac{\tau + \sigma}{\lambda},$$

(the probability that the demonstrator recovers from a given episode of illness) and

$$Ge(h; \psi) = (1 - \psi)^{h-1} \psi, \quad \text{(Geometric)}$$

$$Po(n; \alpha_1 u + \alpha_2 w) = \frac{e^{-(\alpha_1 u + \alpha_2 w)} (\alpha_1 u + \alpha_2 w)^n}{n!}, \quad \text{(Poisson)}$$

$$Ga(u; h, \lambda) = \frac{u^{h-1} e^{-u\lambda} \lambda^h}{\Gamma(h)} \quad \text{(Gamma)}$$

are the relevant probability density/mass functions and where $\Gamma(h)$ is the gamma function.

A.2.1 Cultural Fitness

Using the same approach as before, we can derive an expression for the cultural fitness of the trait in this general model. Noting that $E(H) = 1/\psi$, which is the mean number of episodes,

$$\begin{aligned}\phi = E(N) &= E(E(N|U,W)) = \alpha_1 E(U) + \alpha_2 E(W)\\ &= \alpha_1 E(E(U|H)) + \alpha_2 E(E(W|H))\\ &= \alpha_1 E\left(\frac{H}{\lambda}\right) + \alpha_2 E[(1-q)E(W_{H-1}|H) + qE(W_H|H)]\\ &= \frac{1}{\psi}\frac{\alpha_1}{\lambda} + \left(\frac{1}{\psi} - 1 + \frac{\tau+\sigma}{\lambda}\right)\frac{\alpha_2}{\zeta}. \qquad (5)\end{aligned}$$

This expression of the cultural fitness generalises the two models introduced in the previous section. When there is only a single episode of illness, $\epsilon = 0$ and therefore $\psi = 1$ and ϕ reduces to the previous formula for cultural fitness (Equation 3). With both $\epsilon = 0$ and $\alpha_2 = 0$, the expression simplifies to the cultural fitness value of the initial model (Equation 1). If there are multiple episodes of illness, but demonstration only takes place during periods of illness then $\alpha_2 = 0$, $\epsilon > 0$, and $\phi = \alpha_1/(\psi\lambda)$.

The conversion parameters α_1 and α_2 have straightforward relationships with the cultural fitness, with both elevating the cultural fitness as they increase. The cultural fitness of the treatment is very sensitive to increasing ϵ and α_2 from zero. The former change allows multiple episodes of illness while the second allows demonstration of the treatment during healthy periods. Figures 4 and 5 show this sensitivity for two submodels: 1) single-episode of illness with continued demonstration and 2) multiple episodes of illness with disease restricted demonstration. In the vicinity of parameter values considered here, ϵ and α_2 each has a strong effect on cultural fitness by increasing it dramatically and by creating a high secondary peak at high efficacy values (τ). Decreasing μ or ν lengthens the periods of being sick and being well, respectively, increasing the opportunities for spread of the treatment and therefore its cultural fitness. More complex relationships hold between cultural fitness and the recovery parameters σ, τ and the abandonment parameters ρ, a, because of the trade-offs mentioned in the main text of the article.

A.2.2 Probability of spread

The probability of spread of the behavioural trait in this general model can be considered in two ways. First, consider the probability that the trait spreads beyond the inventor regardless of whether it spreads any further. As before, this probability is given by $P_{sfi} = 1 - P(N = 0)$. Using Equation (4) and noting that

$$\begin{aligned}&\int_0^\infty \int_0^\infty Ga(u;h,\lambda)Ga(w;h-1,\zeta)Po(0;\alpha_1 u + \alpha_2 w)du\ dw\\ &\quad = \int_0^\infty \frac{u^{h-1}\lambda^h e^{-u(\alpha_1+\lambda)}}{h!}du \int_0^\infty \frac{w^{h-2}\zeta^{h-1}e^{-w(\alpha_2+\zeta)}}{(h-1)!}dw\\ &\quad = \left(\frac{\lambda}{\alpha_1+\lambda}\right)^h \left(\frac{\zeta}{\alpha_2+\zeta}\right)^{h-1}\end{aligned}$$

and writing $L = \lambda/(\alpha_1 + \lambda)$ and $M = \zeta/(\alpha_2 + \zeta)$, P_{sfi} simplifies to

$$\begin{aligned}
P_{sfi} &= 1 - \sum_{h=1}^{\infty} \psi(1-\psi)^{h-1}((1-q)L^h M^{h-1} + qL^h M^h) \\
&= 1 - \psi \left(\frac{1-q}{(1-\psi)M} \sum_{h=1}^{\infty}((1-\psi)LM)^h + \frac{q}{1-\psi} \sum_{h=1}^{\infty}((1-\psi)LM)^h \right) \\
&= 1 - \frac{L\psi(1-q+qM)}{1-(1-\psi)LM} \\
&\quad \text{or} \quad \frac{1 - LM + L\psi(1-q)(M-1)}{1 - LM + LM\psi}
\end{aligned}$$

where again $q = (\tau + \sigma)/\lambda$.

This probability of spreading from the inventor can be viewed as an upper bound on the ultimate probability of spread (see also Figure 5c).

A.2.3 Simulation of model

To study the ultimate probability of spread of the treatment in the population we use computer simulations because it is not possible to derive an explicit analytical expression for this probability in the general model. In considering extinction probabilities, a continuous time branching process is equivalent to a discrete time branching process in which all times at which observers are converted by each demonstrator are synchronised. This is possible because the distributions of converts produced by demonstrators are independent and identical, and unaffected by time. The algorithm we use for simulating the model is therefore as follows.

1. Specify parameter values.
2. Set a single sick demonstrator who discovers or invents the treatment. Set number of individuals practising treatment to 1.
3. For each practising individual:
4. Generate exponential time until recovery, death or abandonment with parameter λ. Store this time.
5. Compute whether recovery occurred — with probability $q = (\tau + \sigma)/\lambda$.
6. If recovery occurred, then
   (a) generate exponential time (with parameter ζ) until sickness starts again or death or abandonment occur. Store this time.
   (b) Compute whether demonstrator becomes sick again — with probability ϵ/ζ.
7. Repeat generating exponential times (go to step 4) until death or abandonment occurs. Then add up all the times of being sick (U) and being well (W).
8. Compute $\alpha_1 U + \alpha_2 W$. Generate a Poisson variable with parameter equal to this quantity.

9. If the number of new converts is zero at the first "generation", extinction occurred. Otherwise, track the total number of practising individuals. Return to step 3 in order to repeat the procedure for each practising individual in the population. If the practising population size is beyond some (arbitrary but large) threshold, say fixation has occurred.

10. Repeat the process from step 2 many times and track the proportion of simulations in which extinction occurs.

11. Vary parameters and start again from step 1.

We assume fixation has occurred if the size of the population using the treatment reaches a threshold of 500. The probability of spread is simulated as the proportion of 1000 runs in which fixation occurs.

References

[1] Casella G, Berger RL (2002) Statistical inference. Duxbury, Pacific Grove.

[2] Karlin S, Taylor H (1975) A first course in stochastic processes. New York: Academic Press.
